# Italian validation of the interpersonal needs questionnaire (INQ-15-I) in a sample of university students

**DOI:** 10.1192/j.eurpsy.2021.1559

**Published:** 2021-08-13

**Authors:** S. Magliocca, F. Dolenz, A. Silva, F. Madeddu, R. Calati

**Affiliations:** 1 Psychology, University of Milano-Bicocca, Barlassina, Italy; 2 Psychology, University of Milano-Bicocca, Rapallo (GE), Italy; 3 Psychology, University of Milano-Bicocca, Romanengo, Italy; 4 Psychology, University of Milano-Bicocca, Milan, Italy; 5 Psychiatry, Nîmes University Hospital, Nîmes, France

**Keywords:** Thwarted belongingness, Suicide, Interpersonal-psychological theory, Perceived burdensomeness

## Abstract

**Introduction:**

The Interpersonal Theory of Suicide posits that thwarted belongingness (TB) and perceived burdensomeness (PB) are proximal causes of current suicidal desire, while the acquired capability predicts suicide attempts (Joiner, 2005).

**Objectives:**

This study examined the psychometric properties of the Interpersonal Needs Questionnaire (INQ-15-I), as a measure of the two constructs, on a sample of 458 Italian university students.

**Methods:**

After examining the socio-demographic, clinical and psychosocial characteristics of the sample, we tested the differences in current suicidal ideation and lifetime suicide risk among students. We conducted a confirmatory factor analyses to identify the latent structure of the INQ-15-I and we tested the reliability, criterion concurrent validity and convergent/discriminant validity.

**Results:**

The socio-demographic, clinical and psychosocial features of the sample are in line with the literature on the topic. A two-factor related model with 15 item, showed a good fit to the data and subscales showed excellent internal consistencies (α ≥ 0.84). TB, but mostly PB, were mainly correlated with concurrent suicidal ideation and less with suicidal risk. Divergent validity has emerged with the constructs of the acquired capability. Convergent validity is supported for similar constructs such as depression, low reasons for living, anxiety, interoceptive awareness, psychological pain, loneliness, absence of social support and low self-esteem.

**Conclusions:**

INQ-15-I provides a valid measure of the interpersonal needs among Italian university students by indirectly investigating suicidal risk factors. It also suggest specific clinical intervention areas in suicide prevention. Thus it may be included in suicide risk assessment protocols in Italy.

